# The gap between parental knowledge and children practice of myopia control and challenge under COVID-19: a web-based survey in China

**DOI:** 10.3389/fpubh.2024.1344188

**Published:** 2024-06-12

**Authors:** Hao Yuan, Huibin Lv, Xuemin Li

**Affiliations:** ^1^Department of Ophthalmology, Peking University Third Hospital, Haidian, China; ^2^Beijing Key Laboratory of Restoration of Damaged Ocular Nerve, Beijing, China

**Keywords:** parental knowledge, children’s behaviors, COVID-19, myopia control, refractive status

## Abstract

**Objective:**

To evaluate parental knowledge of myopia control, investigate its association with children’s practice and refractive status, and explore their change under the outbreak of COVID-19 pandemic.

**Methods:**

In this web-based survey, a self-administered questionnaire was made online available during the COVID-19 outbreak between February 1th, 2022 and August 31th, 2022 in China. Participants were recruited via social media by convenience and snowball sampling. Parents of both sexes whose children aged between 3 and 18 were eligible. The overall questionnaire was composed of four categories: demographic information, parental knowledge of myopia, children’s myopia-related behaviors and their change after the COVID-19 pandemic, and children’s refractive status. SPSS version 18.0 was applied to perform the statistics analysis and *p* < 0.05 was considered to be statistically significant.

**Results:**

A total of 423 eligible families were included in our online survey. The average age of children was 11.37 ± 2.83y (male 46.1%; female 53.9%), with a myopia incidence of 83.9% (355/423). Both children’s age (OR = –0.6; 95%CI = –1.12 to −0.07; *p* = 0.026) and family income (OR = 2.60; 95%CI = 1.13 to 4.07; *p* = 0.001) had independently significant impacts on parental knowledge. Unexpectedly, parental knowledge was negatively correlated with children’s onset age of myopia (*p* = 0.002, *r* = −0.165) and positively correlated with spectacles wearing (*p* = 0.014, *r* = 0.131), and no correlation was found between parental knowledge and the occurrence of children myopia, current diopter, annual myopia progression and the diopter of the first glasses (all *p* > 0.05). We found discordance phenomenon between parents’ knowledge and children’s behaviors, with parental knowledge being irrelevant to children’s sleeping time (*p* = 0.159, *r* = 0.069), the frequency of lying reading (*p* = 0.462, *r* = −0.036) and keeping nutrition diet (*p* = 0.142, *r* = 0.072), and positively correlated with daily homework time (*p* = 0.012, *r* = 0.123). After the outbreak of COVID-19, 77.8% (329/423) of parents admitted that their children’s daily routine had been changed, with children spending more time on sleeping (*p* < 0.001) and electronic products (*p* < 0.001), and taking less time to do outdoor activities (*p* < 0.001).

**Conclusion:**

The ideal interaction mode that establishing positive impact between parental knowledge and children practice has not been reached in China, which might be the result of insufficient parents’ cognition and discordance phenomenon between parental knowledge and children’s behaviors. The pandemic of COVID-19 has obviously changed children’s daily routine. More efforts should be made to narrow the gap between knowledge and behaviors of myopia control, and stay alert to the potential increased risk of myopia during COVID-19.

## Why carry out this study?

- The association between parental knowledge of myopia control and children’s refractive status has not been determined in China.
- The impact of COVID-19 pandemic and quarantine on children’s eye health habits has not been explored.

## What was learned from the study?

- The ideal interaction mode that establishing positive impact between parental knowledge and children practice has not been reached in China.
- Discordance was noted between parental knowledge and child behavior.
- The pandemic of COVID-19 has obviously changed children’s daily eye health routine.

## Introduction

With the dramatically increased prevalence in the past three decades, myopia, the most common ocular disorder characterized by a mismatch between ocular optical power and excessive axial length, is arguably reaching epidemic levels (1). It was recognized as a global twenty-first century public health problem and predicted to affect half of the world population by 2050 (2). In China, the prevalence of myopic children aged 7 to 18 years has been reported to increase from 47.5% in 2005 to 57.1% in 2014 (3). Children are becoming myopic at a younger age, with the degree of myopia increasing in magnitude over time (2–4). More than optical inconvenience, pathological myopia could also lead to irreversible vision loss due to serious pathological complications, including retinal tears, retinal detachment and myopic macular degeneration (1, 5). However, currently, the complicated mechanism and pathogenesis of myopia has not been fully elucidated yet, and the efficacy of existing prevention measures is still controversial, contributing to the dilemma of myopia control. Optical correction, which is the primary mode and most widely used treatment option for myopia correction, could not stop the progression of myopia and the occurrence of pathological complications. Therefore, it is necessary to propose more preventive measures to avoid the onset and progression of myopia.

Despite the recent progress in orthokeratology and pharmacologic interventions, it is still a common view that environmental factor plays a dominant role in myopia control practice, and behavior control including less near work time, limited screen time and sufficient outdoor activities strongly affects children refractive status (6). Hence, children’s daily routine behaviors should be put more emphasis on, which can be modified to reduce environmental risk exposure and achieve prevention (7). Traditionally, parents are generally the sole guardians of children, and school-aged children are always inclined to agree to parents’ arrangements (8). Parents can play an integral role given their particular influence on the lifestyle choices of children. Previous studies have revealed that parental knowledge and attitudes had an important influence on children’s physical activities and screen time (9). Therefore, we hypothesized that parental knowledge about myopia control had a significant influence on children’s myopia risk by guiding children’s daily behaviors and rectifying their unhealthy visual habits. The success of any strategy that requires behavioral modification among children, including limited near work duration, encouraged outdoor activity, appropriate lighting environment, timely optical correction and prevention measures, will likely depend on parental awareness of the condition and their acceptance of the proposed interventions.

Thereby, the interaction mode that establishing positive impact between parental knowledge and children practice (PPP mode) was proposed and applied by governments in a few countries for implementing myopia control strategies, including Singapore and Germany (10, 11). However, the high prevalence and its dramatically increasing trend in China indirectly revealed that the ideal PPP mode of myopia control has not been achieved. Thereby, it is essential to investigate the association between parental knowledge and children practice to reveal the “real world” mode of myopia control in China. The association, to the best of our knowledge, has rarely been reported in China.

Moreover, with Corona Virus Disease 2019 (COVID-19) quickly spreading worldwide, governments have imposed unprecedented public measures such as school closure and home confinement to restrict individual contact. According to UNESCO, over 160 countries have closed schools in attempt to retard the spread of COVID-19, covering 87% of world’s student population (12). Consequently, teenagers’ daily routine has been dramatically changed. It has been proposed that the global incidence of myopia would be very likely to increase during the COVID-19 pandemic, provoking the concern of “quarantine myopia,” which might become a secondary epidemic public health problem (13). Hence, it is desperately necessary to explore the alteration of children’s behaviors under the pandemic and stay alert to the potential increased risk of myopia during COVID-19.

Our study aims, therefore, to evaluate parental knowledge of myopia control, investigate its association with children’s practice and refractive status and reveal the “real world” interaction mode between them in China as a means to inform future health planning and policy. Meanwhile, we also explored the change of children’s behaviors under the outbreak of COVID-19 pandemic to remind us of the potential public health problem of “quarantine myopia” and the necessity of strengthening preventive strategies.

## Methods

### Recruitment and inclusion of participants

In this descriptive cross-sectional web-based survey, we developed an electronic survey via the Jinshuju survey platform (AdMaster, Beijing, China) openly accessible to Chinese population. A self-administered questionnaire evaluating parental perception of myopia, children’s myopia-related behavior and current refractive status was made available online between February 26th, 2022 and March 12th, 2022. Before answering any research-related questions, participants would provide the informed consent. The project was approved by the medical ethics committee of Peking University Third Hospital and conducted according to the declaration of Helsinki.

Participants were recruited electronically via social media, mainly through social networking applications. The online convenience and snowball sampling were applied in our survey, and respondents were asked to pass on the survey link to more qualified participants. Parents of both sexes were eligible for inclusion if they could provide complete answers and their children were between 3 and 18 years old at the time of study. To prevent the disturbance of shared views within a family, only one parent from each family was invited to participate in our study. As exclusion criteria, we also excluded incomplete, duplicate, typing error and unrealistic answers before carrying out the analysis. All responses were anonymized at the time of data collection.

### Web-based questionnaire

The structure and content of the web-based survey were designed by a team of expert optometrists and ophthalmologists. The questionnaire was pilot tested during an iterative process to assess the acceptability and feasibility, and subsequently modified until all designers agreed on the final version. The overall questionnaire was composed of four categories: demographic information, parental knowledge of myopia, children’s myopia-related behaviors and their change after the COVID-19 pandemic, children’s refractive status. Additionally, a preceding question was applied at the beginning of the questionnaire to screen out people without children who could also voluntarily provide their perception of myopia as respondents.

The demographic information included the age, educational, occupation, income and refractive status of parents and children’s characteristics such as gender, age, place of residence, daily care people and regular place for visual acuity (4)/optometry examination. The number of children in each family was asked to be provided, and the questions about children should be answered on the basis of the eldest one in each family to avoid potential duplication. And children who stayed with the other family member (not parents) or in boarding schools were also excluded.

Parental knowledge about myopia was estimated by the questions described in the Supplementary materials. To quantitatively analyze parental knowledge, for each question, each participant would obtain 1 score for complete correct answer, 0.5 score for partly correct answer and 0 score for wrong answer. The total score of 16 questions was calculated and converted into 100 scale to assess the overall knowledge of myopia.

Children’s current refractive status was evaluated by the diagnosis of myopia, initial age of myopia, diopter of the first prescribed spectacle, current refractive error (diopter) and annual progression of myopic diopter. The diagnosis of myopia was defined as spherical equivalent (4) of refractive error of less than −0.50 diopters (D) with the cycloplegic refraction. Children’s myopia-related behaviors were assessed by the length of their daily time spend on reading, electronic screens and outdoor activity, the frequency of resting after continuous near work, lying reading, maintaining a distance over 30 centimeters when writing, keeping sufficient light when reading, more than 8-h sleeping, doing Chinese eye exercises and keeping a nutritionally balanced diet. The answers of these questions were divided into five ranks (i.e., always, usually, often, seldom and never) and scored from 1 to 5 according to their contribution of myopia development. Moreover, participants were asked whether their children’s daily routine had been changed and the detailed alterations of children’s behaviors after the COVID-19 pandemic.

Both closed and open-ended questions were used in an adaptive format. The type of answer was determined as single choice or multiple choice depending on the property of questions. The answer options for the multiple-choice questions were randomized to eliminate answer option order bias. No time limitation was set during the completion of the questionnaire by participants.

### Data analysis

SPSS version 18.0 was applied to perform the statistics analysis. Continuous variables in our study were presented as the mean ± SD (standard deviation) and categorical variables were expressed as number and percentage. The chi-square analysis was performed to assess the differences in the characteristic of the categorical variables and t-test analysis for continuous variables. After univariate analysis of potential associations, we performed logistic regression model and multiple linear regression model to identify the independent risk factors for myopia and explore the association between parents’ knowledge and children’s behaviors. The correlation between parental knowledge and children’s myopia status were calculated and adjusted by children age, sex, living place and family income through regression analysis. *p* < 0.05 was considered to be statistically significant. Odds ratios (OR) and 95% confidence intervals (95%CI) were calculated for risk factors that were independently associated with myopia in this population.

## Results

### Demographic information and myopia status

**Figure 1:**
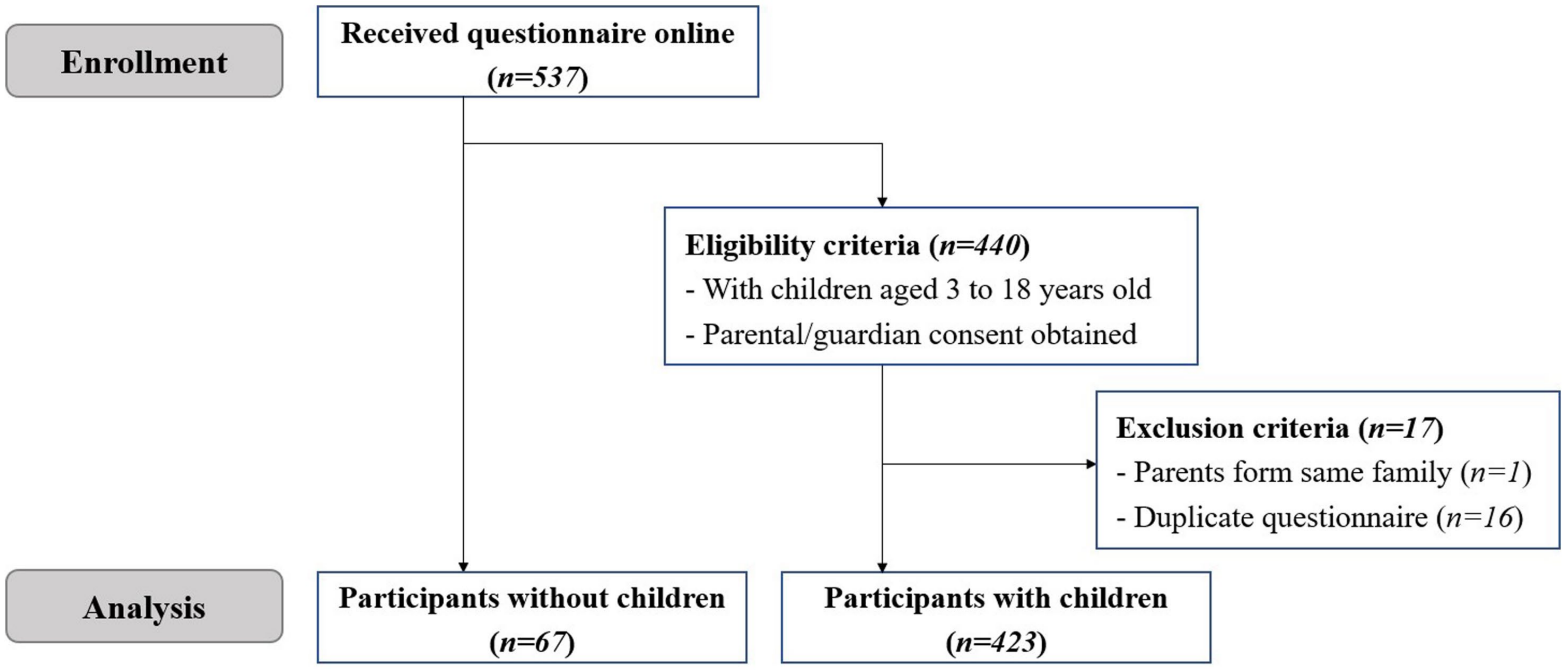
The flow diagram of enrolling and screening eligible participants through the study.

A total of 537 questionnaires were received online in our electronic survey, with 440 parents recruited and 67 participants without children enrolled. The flow of participant enrollment and screening in our study was illustrated in Figure 1. After the exclusion of one parent from the same family and 16 duplicate questionnaires, 423 parents whose children aged from 3 to 18 were eventually included in our analysis.

Table 1 showed the demographic characteristics of involved parents and their children. Among the 423 families, 76.1% (322/423) of them were one-child. 96.9% (410/423) of families lived in the urban area, compared with 3.1% (13/423) in the rural area. In terms of monthly income, more than 80 % of families earned more than 5,000 yuan per person, and 30 % of families made over 10,000 yuan per person. Of these parents, the mean ages of fathers and mothers were 43.44 ± 5.56y and 41.44 ± 4.26y, respectively. More than 80 % of parents’ education background was bachelor degree or above. 80.9% (342/423) of fathers and 70.4% (298/423) of mothers were occupied in mentor labor, with 5.4% (23/423) of fathers and 13.0% (55/423) of mothers engaged in medicine and healthcare work. 68.3% (289/423) of fathers and 76.1% (322/423) of mothers were myopic, and 19.1% (81/423) of fathers and 22.9% (97/423) of mothers were highly myopic (more than −6.00 D). Among the children, 46.1% (195/423) of them were boys and the mean age was 11.37 ± 2.83y. 89.4% (378/423) of children were daily taken care of by their parents and 9.7% (41/423) were looked after by their grandparents. More than nighty percent of children had regular visual acuity or optometry examination in public hospitals (90.3%), optical shops (2.5%), private hospitals (1.3%), and schools (0.3%).

**Table 1:** Demographic information of involved parents and their children.

**Demographic characteristics**		**mean ± SD or *n* (%)**	
***Parental characteristics***			
		Father	Mother
	Age, mean ± SD (y)	43.44 ± 5.56	41.44 ± 4.26
	Education level, *n* (%)		
	Doctor and higher	69 (16.3%)	53 (12.5%)
	Master	133 (31.4%)	130 (30.7%)
	Bachelor	170 (40.2%)	188 (44.4%)
	College	33 (7.8%)	34 (8.0%)
	Technical school or less	18 (4.3%)	18 (4.3%)
	Occupation, *n* (%)		
	Medicine and healthcare	23 (5.4%)	55 (13.0%)
	Physical labor	11 (2.6%)	8 (1.9%)
	Mental labor	342 (80.9%)	298 (70.4%)
	Free occupation	35 (8.3%)	52 (12.3%)
	Else	12 (2.8%)	10 (2.4%)
	Myopia, *n* (%)	289 (68.3%)	322 (76.1%)
	High myopia, *n* (%)	81 (19.1%)	97 (22.9%)
	Children number, *n* (%)		
	One	322 (76.1%)	
	Two	97 (22.9%)	
	Three or more	4 (0.9%)	
	Income, *n* (%)		
	0–2000 yuan/month/person	8 (1.9%)	
	2000–5,000 yuan/month/person	50 (11.8%)	
	5,000–8,000 yuan/month/person	104 (24.6%)	
	8,000–10,000 yuan/month/person	96 (22.7%)	
	>10,000 yuan/month/person	165 (30.0%)	
***Children characteristics***			
	Age, mean ± SD (y)	11.22 ± 3.01	
	Gender, *n* (%)		
	Male	195 (46.1%)	
	Female	228 (53.9%)	
	Place of residence, *n* (%)		
	Urban	410 (96.9%)	
	Rural	13 (3.1%)	
	Daily care people, *n* (%)		
	Patents	378 (89.4%)	
	Grandparents	41 (9.7%)	
	Baby-sitter	2 (0.5%)	
	Him/herself	2 (0.5%)	
	Regular place for optometry examination, *n* (%)		
	Public hospital	287 (90.3%)	
	Private hospital	4 (1.3%)	
	Optical Shop	8 (2.5%)	
	School	1 (0.3%)	
	No regular examination	18 (5.7%)	

Children’s myopia status was presented in Supplementary Table S1. Based on the reports of parents, 83.9% (355/423) of children were once diagnosed as myopia with a diopter of more than 0.50 D. Among these myopic children, the onset of myopia was developed at an average age of 9.02 ± 2.10y. According to the current diopter, 66.8% (237/355) of children were mild myopia (−0.50 D to −3.00 D), 31.5% (112/355) of children were moderate myopia (−3.00 D to −6.00 D) and 1.7% (20/355) of children were high myopia (more than −6.00 D). The annual progression of diopter was less than 1.00 D among three-fourths of myopic children and more than 2.00 D among 10 % of myopic children. 89.6% (318/355) of myopic children wore spectacles, and more than 80 % of them wore their first spectacles with a diopter between −0.50D to −2.00D.

The correlation analysis of demographic information and children’s myopia onset was shown in Supplementary Table S2. The univariate analysis demonstrated that children’s age (11.92 ± 2.43 vs. 7.93 ± 3.21; *p* < 0.001), female (55.7% vs. 39.1%; *p* = 0.034), urban residence (98.5% vs. 89.1%; *p* = 0.003), father’s age (44.18 ± 5.31 vs. 39.67 ± 5.49; *p* < 0.001), and mother’s age (42.15 ± 3.85 vs. 37.61 ± 4.32; *p* < 0.001) showed significant difference between myopia children and non-myopia children, which were subsequently included as possible risk factors in multivariate analysis. By conducting logistic regression model, we detected that increased children’s age (OR = 1.86; 95%CI = 1.51 to 2.30; *p* < 0.001), female children (OR = 2.89; 95%CI = 1.27 to 6.58; *p* = 0.11), urban residence (OR = 16.8; 95%CI = 3.29 to 85.80; *p* < 0.001) and increased mother’s age (OR = 1.198; 95%CI = 1.02 to 1.41; *p* = 0.027) were related to the increased risk of myopia.

### Parental knowledge of myopia

**Figure 2:**
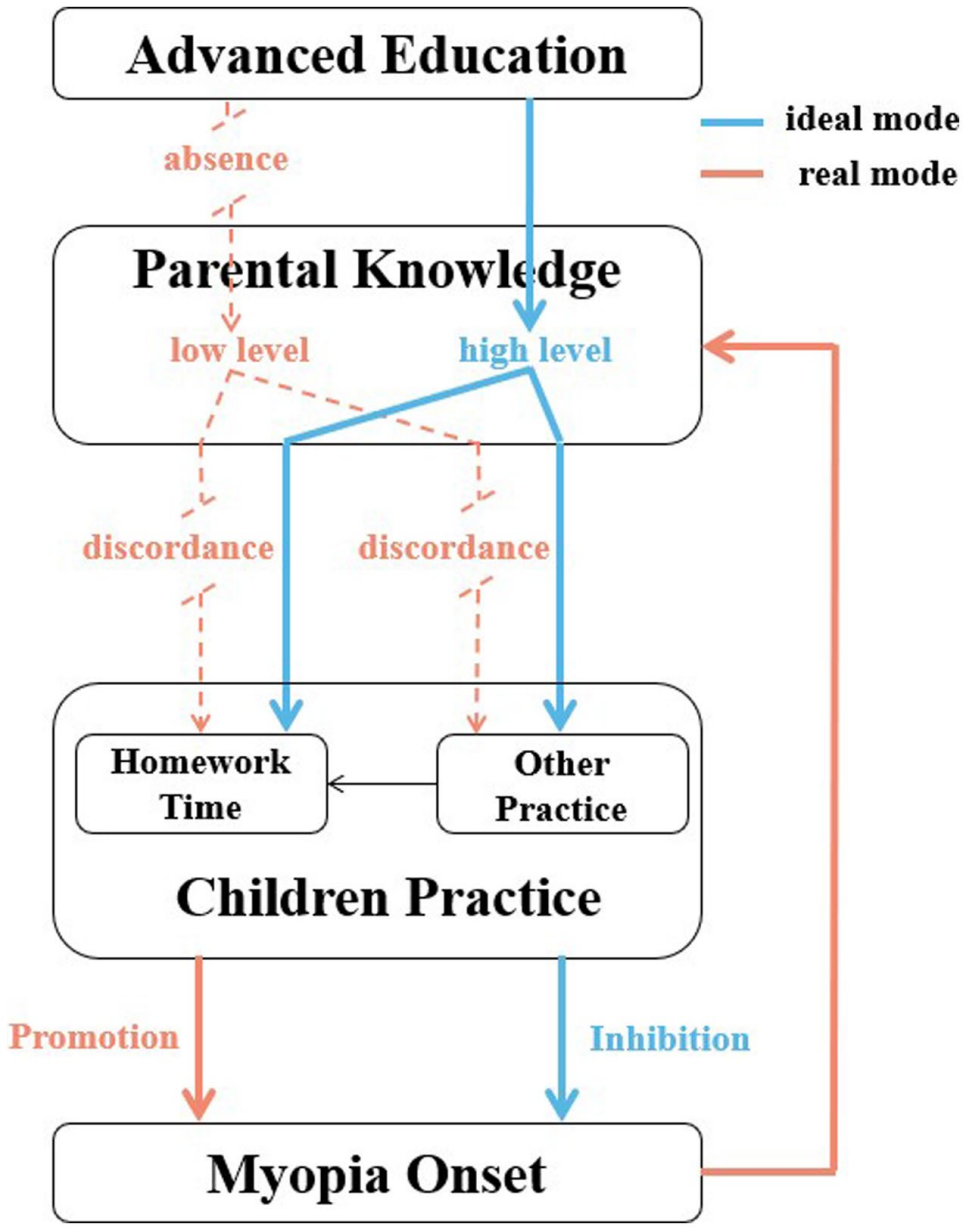
The ideal and realistic interaction mode between Parental Knowledge and Children Practice of Myopia Control in China.

Parents’ answers to myopia knowledge questions were illustrated in Figure 2. The average false rate of these questions was 30.0%. Among them, the false rates of Question 1(41.1%), Question 4 (41.6%), Question 8 (65.7%), Question 10 (70.7%) and Question 13 (63.1%) were over the average value. In terms of the perception of myopia, only 58.9% (249/423) of parents regarded myopia as a pathological disease that could result in a series of severe complications, while 22.7% (96/423) considered only high myopia was pathological and 11.6% (49/423) thought myopia was just a harmless blurred-vision condition. When asked about the correction of myopia, only 58.4% (247/423) of parents thought myopia should be fully corrected, while 27.4% (116/423) held a view that myopia should be under-corrected. Nearly half of parents (203/423, 48.0%) considered that wearing eyeglasses could result in the deformation of eyes, such as “goldfish eyes.” The inhibition effect of low-dose atropine on myopia progression was merely realized among 29.3% (124/423) of parents. Only 74.0% (313/423) of parents were aware that myopia cannot be cured once it occurs, and the others thought myopia was reversible or could be cured by eyeglasses and laser surgery. The detailed distribution of parental answers was shown in Supplementary Table S3. After being converted into one hundred scale, the average score of myopia knowledge among parents was 64.24 ± 16.32, which was significantly higher when compared with that of participants without children (57.35 ± 19.87; *p* = 0.004).

The results of correlation analysis between demographic information and parental knowledge score were tabulated in Supplementary Table S4. In simple linear regression, our analysis showed that children’s age (*p* = 0.009), place of residence (*p* < 0.001), parental education level (both *p* < 0.001), parental occupation (physical labor: *p* = 0.001 for father and *p* = 0.03 for mother; mental labor: *p* = 0.002 for father and *p* = 0.022 for mother; free occupation: *p* = 0.011 for father and *p* = 0.023 for mother), parental myopia status (*p* = 0.001 for father and *p* = 0.044 for mother) and family income (*p* < 0.001) were all significantly correlated with parents’ knowledge. The above variables were subsequently added into the multiple linear regression model, and the results demonstrated that only children’s age (OR = –0.6; 95%CI = –1.12 to −0.07; *p* = 0.026) and family income (OR = 2.60; 95%CI = 1.13 to 4.07; *p* = 0.001) had an independently significant impact on parental knowledge of myopia.

Meanwhile, as shown in Table 2, the relationship of parental myopia knowledge and children’s refractive status was determined by correlation analysis. The results showed that parental knowledge was negatively correlated with children’s onset age of myopia (*p* = 0.002, *r* = −0.165) and positively correlated with spectacles wearing (*p* = 0.002, *r* = 0.230). There was no correlation between parental knowledge and the occurrence of children myopia, current diopter, annual myopia progression and the diopter of the first glasses (all *p*>0.05). Based on the total knowledge score, parents were then divided into high and poor knowledge subgroups with a cut-off value of 60. Similarly, only children’s onset age of myopia (8.81 ± 2.01 vs. 9.29 ± 2.19; *p* = 0.007) and spectacles wearing (92.6% vs. 85.6%; *p* = 0.034) showed significant difference between the two groups.

**Table 2:** The correlation analysis between parental knowledge and children’s myopia status.

**Children’s myopia status**		**Correlation with parental knowledge**		**Difference between high and poor knowledge subgroups**
		***p*-value**	***r* value**	***p*-value**
	Myopia	0.327	−0.077	0.191
	Onset age of myopia	0.002*	−0.165	0.007^#^
	Current diopter	0.296	0.056	0.250
	Spectacles wearing	0.002*	0.230	0.034^#^
	Diopter of the first glasses	0.642	−0.026	0.652
	Annual progression	0.434	0.044	0.237

* Significantly correlated with parental knowledge adjusted by children age, sex, living place and family income (*p* < 0.05). ^#^ Significantly different between high and poor knowledge subgroups (*p* < 0.05).

### Children’s behaviors

The information of children’s myopia-related behaviors was shown in Supplementary Table S5. According to the results, more than nighty percent (91.0%) of children could sleep at least 7 h a night. However, only less than 20% (19.1%) of children could finish their homework within 1 h, and nearly half of them spend more than 4 h on homework. Additionally, more than 2 h of daily outdoor activities could be ensured in just 3.5% (15/423) of children, and half of children (51.5%) took at least 1 h in electronic products. On the other hand, only 8.3% (35/423) and 5.2% (22/423) of children reported “always” for their daily frequency of “taking a break after using eyes continuously for 40 min” and “keeping the distance between eyes and books over 30 centimeters when reading,” respectively.

The correlation analysis between parental myopia knowledge and children’s behaviors were presented in Table 3. In the prevention of developing myopia, parental knowledge was correlated with increased outdoor activities time (*p* = 0.028, *r* = 0.107) and decreased time on electronic products (*p* < 0.001, *r* = −0.191). In addition, high parental knowledge was positively related to children’s frequency of resting their eyes (*p* < 0.001, *r* = 0.193), keeping enough distance between eyes and books (*p* = 0.462, *r* = −0.036), keeping bright environment (*p* = 0.031, *r* = 0.106) and doing Chinese exercises (*p* = 0.002, *r* = 0.150). However, our results showed that parental knowledge was irrelevant to children’s sleeping time (*p* = 0.159, *r* = 0.069) and their frequency of lying reading (*p* = 0.462, *r* = −0.036) and keeping nutrition diet (*p* = 0.142, *r* = 0.072). Moreover, on the contrary, higher parental knowledge was correlated with more daily time on doing homework (*p* = 0.012, *r* = 0.123). Similarly, by comparing high and poor knowledge groups, we found that sleeping time (*p* = 0.666) and the frequency of lying reading (*p* = 0.584) and keeping nutrition diet (*p* = 0.091) showed no significant difference between different levels of knowledge, and high knowledge group spent more time on homework instead (*p* = 0.014).

**Table 3:** The correlation analysis between parental knowledge and children’s behaviors of myopia control.

**Children’s behaviors of myopia control**		**Correlation with parental knowledge**		**Difference between high and poor knowledge subgroups**
		***p*-value**	***r* value**	***p*-value**
	Daily time spent on			
	Sleeping	0.159	0.069	0.666
	Doing homework	0.012*	0.123	0.014^#^
	Outdoor activities	0.028*	0.107	0.034^#^
	Electronic products	<0.001*	−0.191	<0.001^#^
	Daily frequency of			
	Taking a break after using eyes continuously for 40 min	<0.001*	0.193	0.005^#^
	Lying down to do homework and read books	0.462	−0.036	0.584
	Keeping the distance between eyes and books over 30 centimeters when reading	0.031*	0.106	0.079
	Keeping enough bright light when reading and writing	0.015*	0.119	0.125
	Doing Chinese eye exercises	0.002*	0.150	0.006^#^
	Keeping a balanced nutrition diet	0.142	0.072	0.091

* Significantly correlated with parental knowledge (*p* < 0.05). ^#^ Significantly different between high and poor knowledge subgroups (*p* < 0.05).

The results of correlation analysis between children’s behaviors and their myopia status were tabulated in Supplementary Table S5. The univariate analysis demonstrated that sleeping time (*p* = 0.002), homework time (*p* < 0.001), the frequency of resting eyes (*p* < 0.001), keeping enough distance between eyes and books (*p* < 0.001) showed significant difference between myopia children and non-myopia children, which were subsequently added as possible risk factors into multivariate analysis. By conducting logistic regression model, with the adjustment of children’s age, gender, place of residence and mother’s age, we detected that only increased homework time was a significantly independent risk factor of developing myopia (OR = 1.61; 95%CI = 1.01 to 2.58; *p* = 0.046).

With the outbreak of COVID-19, 77.8% (329/423) of parents admitted that their children’s daily routine had been changed due to home quarantine, restricted traffic and online education. Compare with daily behaviors, as shown in Table 4, children spent more time on sleeping (*p* < 0.001) and electronic products (*p* < 0.001), and take less time to do outdoor activities (*p* < 0.001) during the pandemic. Nevertheless, daily time spent on homework did not change significantly after the outbreak (*p* = 0.092). The correlation analysis demonstrated that basic demographics and the change of daily routine were irrelevant (all *p* > 0.05). However, parental knowledge of myopia was positively correlated with the change of daily routine (*p* = 0.002, *r* = 0.148), and high knowledge group inclined to change their daily routine during the pandemic (81.0% vs. 72.1%; *p* = 0.033). We then performed the subgroup analysis based on parental knowledge in Supplementary Table S6. The results showed that homework time also remarkably lengthened in high knowledge group (*p* = 0.034), which did not significantly change in poor knowledge group (*p* = 0.657).

**Table 4:** The comparison of children’s behaviors before and after the outbreak of COVID-19.

**Children’s behaviors**	**Routine**	**During pandemic**	***p*-value**
Daily time spent on			
Sleeping			<0.001*
<7 h	35 (10.6%)	20 (6.1%)	
7–8 h	116 (35.3%)	51 (15.5)	
8–9 h	127 (38.6%)	107 (32.5)	
9–10 h	46 (14.0%)	105 (31.9)	
>10 h	5 (1.5%)	46 (14.0)	
Doing homework			0.092
<1 h	16 (4.9%)	20 (6.1%)	
1–2 h	42 (12.8%)	49 (14.9%)	
2–4 h	112 (34.0%)	108 (32.8%)	
4–8 h	100 (30.4%)	104 (31.6%)	
>8 h	56 (17.0%)	48 (14.6%)	
Outdoor activities			<0.001*
<0.5 h	82 (24.9%)	256 (77.8%)	
0.5–1 h	125 (38.0%)	43 (13.1%)	
1–1.5 h	80 (24.3%)	19 (5.8%)	
1.5–2 h	34 (10.3%)	9 (2.7%)	
>2 h	8 (2.4%)	2 (0.6%)	
Electronic products			<0.001*
<0.5 h	63 (19.1%)	14 (4.3%)	
0.5–1 h	102 (31.0%)	31 (9.4%)	
1–1.5 h	57 (17.3%)	50 (15.2%)	
1.5–2 h	38 (11.6%)	49 (14.9%)	
>2 h	67 (20.4%)	185 (56.2%)	

* Significantly different after the outbreak of COVID-19 pandemic (*p* < 0.05).

## Discussion

With the sharp rise of prevalence, myopia “epidemic” is becoming a worldwide public health issue, which is considered to be related with both genetic and behavioral factors (1). Generally, given that parents are main child guardians, children’s daily routine and behaviors are largely subject to their parents’ arrangements (8). Therefore, we reasonably hypothesized that parental knowledge of myopia might be associated with children’s behaviors and have a significant influence on children’s myopia risk. Prevention measures for myopia control about behavioral modification will likely require parental precise knowledge and their acceptance of the proposed interventions, depending on the positive feedback mode between parental knowledge and children practice. Additionally, due to school closures and home confinement under the outbreak of COVID-19, children tend to spend longer screen time on online courses and less spare time on outdoor activities, which probably raises the incidence of myopia during COVID-19 pandemic (12). Thus, we conducted this web-based survey through online questionnaires to describe parental knowledge of myopia control, investigate the “real world” interaction mode between parental knowledge and children practice in China, and explore the change of children’s daily routine after the outbreak of COVID-19.

To the best of our knowledge, this is the first research to investigate the effect of parental knowledge about myopia risk on children’s behaviors and myopia status in China. Our findings revealed that, unexpectedly, parental knowledge did not play a significantly positive role in myopia control, which might be the result of discordance phenomenon between parental knowledge and children’s behaviors. Therefore, more efforts should be made to narrow the gap between parents’ knowledge of myopia control and children’s behaviors, thereby effectively establishing “PPP mode” and preventing the occurrence of myopia. Moreover, COVID-19 pandemic has obviously changed children’s daily routine in China. Children inclined to spend more time on sleeping and electronic products, and take less time to do outdoor activities during home confinement. Therefore, it’s necessary to stay alert to the potential increased risk of myopia during COVID-19 and institute preventive measures against “quarantine myopia.”

According to the logistic regression model, increased children’s age, female sex, urban residence and increased mother’s age were related to the risk of myopia. In agreement with previous studies, we found that myopia was more common in older children. McCrann et al. demonstrated that, for older children, increasing academic burden and social contact were followed by more time spent on near work and visual displays coupled with a decline in outdoor time, thereby exerting both a direct and indirect influence on myopia development and progression (8). Meanwhile, in the present study, we found that female sex was associated with a higher risk of myopia, which was in accordance with several previous studies. Lyu et al. speculated that estrogen could regulate matrix metalloproteinase in the sclera and counted for myopia development in female adolescents (14). Apart from the physiological reason, another possible explanation could be that young girls always spend less time engaged in outdoor activities (14). Additionally, our result also verified that urbanization was linked to increasing prevalence of myopia. Urbanization is usually considered to be accompanied by lack of green spaces, less time outdoors, variable light exposure and changes in residents’ lifestyle, diet, and stress in more densely populated areas, all of which could contribute to myopia development. Interestingly, although few studies have found the association between mother’s age and children’s myopia status, our research revealed that children were exposed to higher myopia risk with increasing mother’s age. Similarly, a large-sample cohort study conducted in British by Rahi et al. concluded that, myopia was positively associated with greater maternal age, indicating the fundamental importance of prenatal influences on the ocular growth, and the excessive elongation of myopic eyes might be determined *in utero* and amplified or cascaded during childhood (15). This unexpected association also suggested we should keep in mind that the trend of increasing births to older mothers, which had been identified as a novel putative risk factor of child health and growth, was consistent and possibly related with the global trends of increasing myopia, and further research could explore the potential impact of prenatal and early life factors on the abnormal ocular growth.

As the integral targets of numerous behavioral interventions, parents play an essential role in various aspects of myopia prevention, including the environments to which children are exposed, parenting styles, children’s daily routine and behaviors, all of which were deemed critical in myopia management (8). Therefore, parental awareness of myopia causes and prevention strategies is of much importance in intervening and supervising their children’s behaviors, thereby effectively decreasing the occurrence of myopia. Overall, in our survey, with an average false rate of 30.0%, parental knowledge of myopia did not reach the ideal level. However, compared to participants without children, parents in our survey showed significantly higher level of knowledge, suggesting that parents might be eager to acquire related knowledge about myopia control and seek effective measures to slow myopic progression. We detected most of parents were lacking knowledge about the pathology, irreversibility and intervention strategies of myopia. Myopia is characterized by typical degenerative changes in the sclera, choroid, and retinal pigment epithelium, which leads to elongated axis length, posterior staphyloma and choroidal neovascularization and also occurs in non-highly myopic eyes (5). Nevertheless, when asked about the perception of myopia, a number of parents only regarded myopia as an optical inconvenience instead of a pathological disease with visually threatening complications, and some of them thought myopia was reversible or could be cured by eyeglasses and laser surgery. The fail to realize its pathogenicity and irreversibility could delay regular optometry and ophthalmic examination and medical treatment, which might promote the progression of myopia and occurrence of complications. Moreover, nearly half of parents considered that wearing eyeglasses could result in the deformation of eyes, such as “goldfish eyes” that is actually the result of exophthalmos due to excessive elongated axial length. This wrong perception might reduce parental compliance of wearing children spectacles and accelerate myopia progression. On the other hand, atropine, a nonselective muscarinic antagonist, has been proved effective in several long-term cohort studies in preventing worsening of myopia in children (6). However, due to the uncertainty of exact mechanism and action site, safety concerns such as photophobia, poor near visual acuity and allergy are generally considered to be a barrier for the commercialization and clinical use of atropine in China, which could possibly explain the low awareness rate of the inhibition effect of atropine on myopia progression among parents in our research. A meta-analysis conducted by Ahnul et al. demonstrated that the adverse effects of atropine were dose dependent and the low dose of atropine (0.01%) seemed to decrease the adverse effects (16). Hence, we suggested that a large-scale clinical trial should be investigated further to explore a safety dose with enough efficacy in China. Furthermore, either pharmacological or optical, the role of parents in the acceptance of any interventional treatments needs to be recognized to make them practicable.

According to the multiple regression analysis, parents with younger children might be more deficient in knowledge about myopia control. However, it has been reported that the preschool period is crucial for the development of eyesight, implying that school-aged children would have a lower risk of myopia if parental knowledge can be promoted during preschool period (17). Therefore, it is necessary to strengthen health education for parents of young preschool children to avoid irreversible visual impairment. On the other hand, based on our results, low-income families tend to lack knowledge of myopia control, which suggested that both individual as well as community level socioeconomics play a role in determining parental knowledge. Thus, we propose that regular optometry examinations should be popularized and performed based on community hospitals. Moreover, there might be a relatively large difference in knowledge between fathers and mothers. However, due to the limitation of research design and data collection, the gender of participations who filled out the questionnaire was not acquired, which is needed to be corrected in the future study as a potential confounding variable.

Generally, it is believed that better knowledge and perception have a significant positive influence on the uptake of preventive measures and bridging the gap towards delivering of health information, reducing children’s myopia risk by guiding children’s daily behaviors and rectifying their unhealthy visual habits (9, 18). Nevertheless, our results revealed that higher parental knowledge failed to reduce the occurrence and slow the progression of myopia. On the contrary, parental knowledge was negatively correlated with children’s onset year of myopia and positively correlated with spectacles wearing, suggesting that early-onset myopia and spectacles wearing attracted parental attention instead and enhanced their knowledge in turn by negative feedback. Another possible explanation for the earlier occurrence of myopia in highly cognitive parents could be that they were more aware of the hazards and thus took their children for eye assessments at an earlier age. On the other hand, we also detected that parental knowledge was at odds with several children’s myopia-related behaviors, with parental knowledge being irrelevant to children’s sleeping time, the frequency of lying reading and keeping nutrition diet, and even positively correlated with daily homework time. Therefore, we suspected that the discordance phenomenon between parental knowledge and children’s behaviors might lag the influence of knowledge and contribute to the inconsistency between parents’ knowledge and children’s myopia status. There might be several reasons for the dissociation. First, according to the “KAP (knowledge-attitude-practice)” theory, attitude is a prominent intermediate factor between knowledge and practice, which could directly promote behavioral changes (18). However, in our current study, parents’ attitude was not assessed and their knowledge might fail to be sufficiently transferred to their attitude thoroughly. On the one hand, positive attitude is mainly driven by the high level of knowledge and perceptions toward the harmfulness of myopia. Based on our results, although the overall level of parental knowledge of myopia control was moderate, a substantial part of parents had not recognized the pathology and irreversibility of myopia, thereby failing to consider myopia as a health risk and actively intervene children’s daily behaviors. On the other hand, although parents might have a relatively higher level of knowledge regarding myopia control and prevention, other intrinsic factors which were beyond the assessment of our research, might modify parental attitude toward myopia control and affect their final arrangements in children’s behaviors. These intrinsic factors, including the confidence in the knowledge, the expectation on children’s vision and the concern about the impact of prevention strategies on children’s academic, vary individually and may be related to how the knowledge was received, processed, perceived and interpreted. Hence, we suggested that only enhancing parental knowledge is not enough to improve children’s behaviors. More efforts should be paid to help parents form a distinct perception of the harm of myopia and turn their attitudes by emphasizing the threat of myopia to visual health and increasing the acceptability of prevention measures. Parents’ attitude toward myopia control should be evaluated in the further research and addressed as part of the strategies for myopia prevention. Secondly, with the increase of age, children gradually step into their adolescence and tend to get more involved in schools and society, and their behaviors will consequently be more affected by their teachers, classmates and themselves instead of parents. In that case, more other variables, such as health education in school, teachers’ supervision, classmates’ habits and their own attitudes, will account for their daily behaviors which parental knowledge cannot completely predict. Given the influence schools and society have on children’s lifestyle choices, these findings confirm that school supervision and public education about myopia are essential to generate a shift in individual behaviors. Teachers, schools and society are supposed to take their responsibility together to close the critical gap between parental knowledge and children’s behaviors. However, during the COVID pandemic, the discordance could also be due to some quarantine-related situations where parents have to permit their children to use electronic devices to remain indoors. Therefore, in the future study, the discordance phenomenon should be verified in this post-COVID era.

Ideally, as shown in Figure 2, the ideal interaction mode that establishing positive impact between parental knowledge and children practice was most conducive to the implementation of myopia control strategies. The mode that better children practice derived from higher-level parental knowledge could inhibit myopia development has been widely applied in a few countries. In Singapore, by conducting the visual health screening, designing brochures and performing lectures about myopia knowledge, Singapore National Eye Center allied with schools and media to provide advanced education for parents to enhanced parental cognition level upstream, which has successfully disciplined children’s behavior and decreased the incidence of myopia by 5% in the past 6 years (19). And Germany advocated the specially-designed application software for parents to communicate the risk and harm of myopia, encouraging them to restrict children’s screen time and establish refraction development profile for children (11). However, our research reveals that the ideal positive mode has not been reached in China. Without enough cognition, the positive influence of parental knowledge on children’s behavior has not been facilitated. The present state is that the worsening myopia in turn enhances parents’ knowledge by negative feedback. Based on our results, we consider that the main problems are as follows: firstly, the actively-advanced education for parental knowledge is almost absent, and the passively-enhanced knowledge driven by exacerbated myopia lags behind children’s key period of visual development; secondly, the level of cognition is not enough, especially for the irreversible and pathological characteristic of myopia; thirdly, the discordance phenomenon that parents’ knowledge fails to be sufficiently transferred to children behavior completely. Therefore, we suggest that, in the future, parental knowledge should be actively strengthened upstream with the multi-channel approach, combining parents, teachers, schools, society, ophthalmologists and children themselves together. Myopia knowledge should be more popularized, especially in low-income and rural residents. More efforts should be paid to help parents turn their attitudes by emphasizing the threat of myopia to visual health.

During COVID-19 pandemic, less physical activity, longer screen time and irregular sleep pattern are very likely to increase the incidence of shortsightedness and contribute to the “quarantine myopia epidemic” (12, 13). According to our results, after the outbreak of COVID-19, 77.8% of parents admitted that their children’s daily behaviors had been changed, with more time spent on sleeping and electronic products, and less outdoor activities. More sleep time is considered to be linked to less near work and screen time, and Jee et al. revealed an inverse relationship between sleep duration and myopia (20). Although our survey revealed the prolongation of sleep time, however, we did not explore the alteration of sleep rhythm. Due to the uncertainty of online class time and lack of school restrictions, children might have a disposition to get up and stay up late. Therefore, despite the longer sleep duration, the irregular sleep pattern and circadian rhythms cannot be ignored. Not only sufficient sleeping time but also regular sleep rhythms should be guaranteed to prevent children from developing myopia during the quarantine. Additionally, with the wide application of online classes, children get more access to electronic devices to read, write and finish their homework, which increases screen time, prolongates near work and limits outdoor time. Therefore, we suggested more break time between classes to allow children to rest their eyes and reduce accommodative stimulation. Meanwhile, when the usage of electronic screen devices is unavoidable, more attention could be paid to adjusting devices’ parameters, including luminance ratio, brightness, contrast and softness which were associated with myopia. It is necessary to stay alert to the potential public health problem of “quarantine myopia” and strengthen preventive strategies focusing on children’s visual habits. Meanwhile, post-pandemic myopia evaluation project and ophthalmological surveillance program for children are probably required. Patient education, including theoretical knowledge, practical experience, enhanced compliance and regular follow-up, are all of great importance.

There were several limitations in our research. Firstly, due to online platform and non-randomized sampling, participants in our research were preselected. For instance, urban participants might have more access to the social application and were more likely to participate our survey, and parents with myopia children would be more motivated to complete our questionnaire, which could contribute to potential selection bias. Selection bias may overestimate parents’ mastery of myopia-related knowledge and affect subsequent correlation and regression analyses. Random selection strategy would be a better option for future studies. Secondly, children’s behaviors were obtained from questionnaires and relied on reports of their parents, which could be subject to recall bias. Additionally, as a cross-sectional study, the cause or effect of parents’ knowledge and children’s behaviors toward myopia development was not clear. Therefore, a further cohort study with a larger sample size is required to give a better understanding of parental knowledge about myopia control and demonstrate its real causal relationship with children’s behaviors and myopia risk.

## Conclusion

The ideal interaction mode that establishing positive impact between parental knowledge and children practice has not been reached in China, which might be the result of insufficient parents’ cognition and discordance phenomenon between parental knowledge and children’s behaviors. The pandemic of COVID-19 has obviously changed children’s daily routine. More efforts should be made to narrow the gap between knowledge and behaviors of myopia control, and stay alert to the potential increased risk of myopia during COVID-19.

## Data availability statement

The raw data supporting the conclusions of this article will be made available by the authors, without undue reservation.

## Ethics statement

The studies involving humans were approved by the project was approved by the medical ethics committee of Peking University Third Hospital and conducted according to the declaration of Helsinki. The studies were conducted in accordance with the local legislation and institutional requirements. The participants provided their written informed consent to participate in this study.

## Author contributions

HY: Writing – original draft. HL: Writing – original draft. XL: Writing – review & editing.
