# Supplementary material for: The gap between parental knowledge and children practice of myopia control and challenge under COVID-19: a web-based survey in China
Source: Front Public Health. 2024 Jun 12;12:1344188. doi: 10.3389/fpubh.2024.1344188 (PMC11199406; doi:10.3389/fpubh.2024.1344188)
Supplement: Supplementary file 1 [file Data_Sheet_1.docx]

***Supplementary Figure 1***. Parents’ answers about myopia knowledge questions.


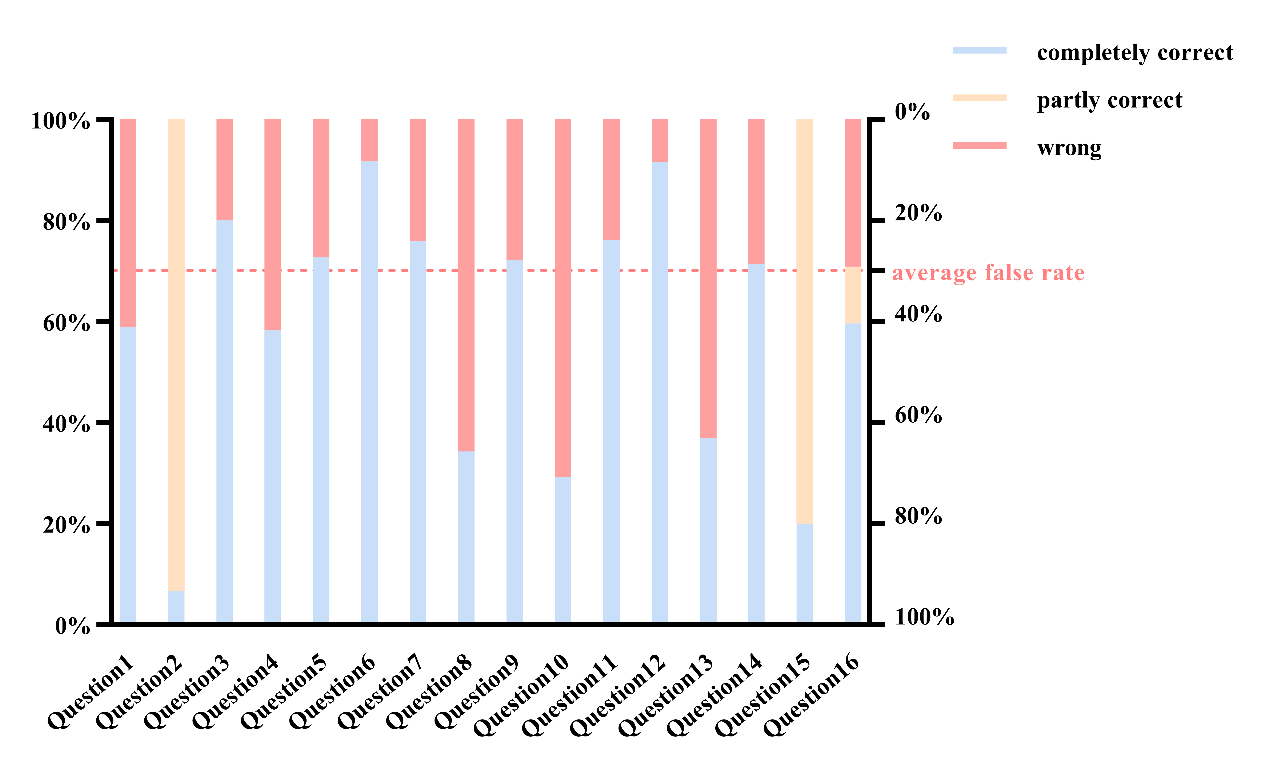


***Supplementary Table 1***. Information of children’s myopia status.

| **Myopia status** | **mean ± SD or n (%)** |
| --- | --- |
| Average age of onset of myopia (y) | 9.02±2.10 |
| Current myopic diopter, n (%) |  |
| 0.50 to 1.00 D | 48 (13.5%) |
| 1.00 to 2.00 D | 101 (28.5%) |
| 2.00 to 3.00 D | 88 (24.8%) |
| 3.00 to 4.00 D | 56 (15.8%) |
| 4.00 to 5.00 D | 42 (11.8%) |
| 5.00 to 6.00 D | 14 (3.9%) |
| more than 6.00 D | 6 (1.7%) |
| Annual progression of myopic diopter, n (%) |  |
| less than 0.50 D/year | 119 (37.4%) |
| 0.50 to 1.00 D/year | 127 (39.9%) |
| 1.00 to 2.00 D/year | 33 (10.4%) |
| 2.00 to 3.00 D/year | 1 (0.3%) |
| 3.00 to 4.00 D/year | 38 (11.9%) |
| Spectacles wearing, n (%) |  |
| Yes | 318 (89.6%) |
| No | 37 (10.4%) |
| Diopter of the first spectacle, n (%) |  |
| less than -0.50 D | 7 (2.2%) |
| -0.50 to -1.00 D | 79 (24.8%) |
| -1.00 to -1.50 D | 111 (34.9%) |
| -1.50 to -2.00 D | 67 (21.1%) |
| more than -2.00 D | 54 (17.0%) |

***Supplementary Table 2.*** The correlation analysis of demographic information and children’s myopia onset.

| **Demographics** | **Myopia group** | **Non-myopia group** | **P value**  **for univariate analysis** | **P value**  **for logistic analysis** | **OR** | **95%CI** |
| --- | --- | --- | --- | --- | --- | --- |
| Children’s age | 11.92±2.43 | 7.93±3.21 | <0.001* | <0.001* | 1.86 | 1.51 to 2.30 |
| Children’s gender |  |  |  |  |  |  |
| Male | 152(44.3%) | 28(60.9%) | 0.034* | 0.011* | 2.89 | 1.27 to 6.58 |
| Female | 191(55.7%) | 18(39.1%) |  |  |  |  |
| Children’s number |  |  |  |  |  |  |
| One | 276 (77.7%) | 31 (66.0%) | 0.137 |  |  |  |
| Two | 76 (21.4%) | 15 (31.9%) |  |  |  |  |
| Three or more | 3 (0.8%) | 1 (2.1%) |  |  |  |  |
| Place of residence |  |  |  |  |  |  |
| Urban | 338(98.5%) | 41(89.1%) | 0.003* | 0.001* | 16.8 | 3.29 to 85.80 |
| Rural | 5(1.5%) | 5(10.9%) |  |  |  |  |
| Daily care people |  |  |  |  |  |  |
| Patents | 313(91.3%) | 36(78.3%) | 0.06 |  | | |
| Grandparents | 26(7.6%) | 10(21.7%) |  |  |  |  |
| Baby-sitter | 2(0.6%) | 0(0%) |  |  |  |  |
| Him/herself | 2(0.6%) | 0(0%) |  |  |  |  |
| Father’s age | 44.18±5.31 | 39.67±5.49 | <0.001* | 0.071 | 0.905 | 0.81 to 1.01 |
| Mother’s age | 42.15±3.85 | 37.61±4.32 | <0.001* | 0.027* | 1.198 | 1.02 to 1.41 |
| Father’s education |  |  |  |  |  |  |
| Doctor and higher | 63(18.4%) | 7(6.5%) | 0.122 |  | | |
| Master | 108(31.5%) | 17(37.0%) |  |  |  |  |
| Bachelor | 138(40.2%) | 19(41.3%) |  |  |  |  |
| College | 23(6.7%) | 4(8.7%) |  |  |  |  |
| Technical school or less | 11(3.2%) | 3(6.5%) |  |  |  |  |
| Mother’s education |  |  |  |  |  |  |
| Doctor and higher | 46(13.4) | 5(10.9) | 0.797 |  | | |
| Master | 103(30.0) | 18(39.1) |  |  |  |  |
| Bachelor | 156(45.5) | 17(37.0) |  |  |  |  |
| College | 27(7.9%) | 2(4.3%) |  |  |  |  |
| Technical school or less | 11(3.2%) | 4(8.7%) |  |  |  |  |
| Father’s occupation |  |  |  |  |  |  |
| Medicine and healthcare | 14(4.1%) | 5(10.9%) | 0.127 |  | | |
| Physical labor | 8(2.3%) | 2(4.3%) |  |  |  |  |
| Mental labor | 294(85.7%) | 35(76.1%) |  |  |  |  |
| Free occupation | 27(7.9%) | 4(8.7%) |  |  |  |  |
| Mother’s occupation |  |  |  |  |  |  |
| Medicine and healthcare | 36(10.5%) | 11(23.9%) | 0.055 |  | | |
| Physical labor | 6(1.7%) | 1(2.2%) |  |  |  |  |
| Mental labor | 259(75.5%) | 27(58.7%) |  |  |  |  |
| Free occupation | 42(12.2%) | 7(15.2%) |  |  |  |  |
| Myopic father | 237(69.1%) | 28(60.9%) | 0.312 |  | | |
| Myopic mother | 262(76.4%) | 35(76.1%) | 0.964 |  | | |
| Income |  |  |  |  |  |  |
| 0-2000 yuan/month/person | 5(1.5%) | 1(2.2%) | 0.416 |  | | |
| 2000-5000 yuan/month/person | 25(7.3%) | 10(21.7%) |  |  |  |  |
| 5000-8000 yuan/month/person | 93(27.1%) | 8(17.4%) |  |  |  |  |
| 8000-10000 yuan/month/person | 84(24.5%) | 8(17.4%) |  |  |  |  |
| ＞10000 yuan/month/person | 136(39.7%) | 19(41.3%) |  |  |  |  |

*: significantly correlated with children’s myopia status (p<0.05)

***Supplementary Table 3.*** The distribution of parental answers about myopia knowledge questions.

| **Questions and Answers** | **Frequency (n, %)** |
| --- | --- |
| Q1: Which is your perception of myopia? |  |
| Myopia is only a blurred-vision condition with no harm to the eyes. | 49 (11.6%) |
| *Myopia is a pathological disease leading to a series of severe complications. | 249 (58.9%) |
| Only high myopia is a pathological disease. | 96 (22.7%) |
| Unclear | 29 (6.9%) |
| Q2: Which are the potential complications of myopia? |  |
| *Retinal tear | 153 (36.2%) |
| *Retinal detachment | 298 (70.4%) |
| *Retinal degeneration | 230 (54.4%) |
| *Retinal hemorrhage | 161 (38.1%) |
| *Glaucoma | 133 (31.4%) |
| *Complicated cataract | 96 (22.7%) |
| *Strabismus | 157 (37.1%) |
| Q3: What time do you think myopia children should wear eyeglasses? |  |
| *Once myopia happens | 339 (80.1%) |
| Until the visual acuity declines dramatically | 45 (10.6%) |
| Unclear | 39 (9.2%) |
| Q4: What extent should myopia be corrected to? |  |
| Under correction | 116 (27.4%) |
| *Full correction | 247 (58.4%) |
| Over correction | 7 (1.7%) |
| Unclear | 53 (12.5%) |
| Q5: Whether mild myopia children should wear eyeglasses? |  |
| *Yes | 33 (7.8%) |
| No | 308 (72.8%) |
| Unclear | 82 (19.4%) |
| Q6: Whether mydriasis agents should be administrated when prescription? |  |
| *Yes | 15 (3.5%) |
| No | 388 (91.7%) |
| Unclear | 20 (4.7%) |
| Q7: Whether wearing eyeglasses could contribute to myopia progression? |  |
| Yes | 37 (8.7%) |
| *No | 321 (75.9%) |
| Unclear | 65 (15.4%) |
| Q8: Whether wearing eyeglasses could result in the deformation of eyes like “goldfish eyes”? |  |
| Yes | 203 (48.0%) |
| *No | 145 (34.3%) |
| Unclear | 75 (17.7%) |
| Q9: Whether the eyeglasses could be worn only when reading instead of all the time? |  |
| Yes | 60 (14.2%) |
| *No | 305 (72.1%) |
| Unclear | 58 (13.7%) |
| Q10: Whether the progression of myopia could be inhibited by low-dose atropine? |  |
| *Yes | 124 (29.3%) |
| No | 44 (10.4%) |
| Unclear | 153 (36.2%) |
| Never heard of atropine | 102 (24.1%) |
| Q11: Whether the progression of myopia could be inhibited by rigid gas permeable contact lens? |  |
| *Yes | 322 (76.1%) |
| No | 14 (3.3%) |
| Unclear | 58 (13.7%) |
| Never heard of rigid gas permeable contact lens | 29 (6.9%) |
| Q12: Whether the onset of myopia could be prevented by daily 2-hour outdoor activities? |  |
| *Yes | 387 (91.5%) |
| No | 5 (1.2%) |
| Unclear | 31 (7.3%) |
| Q13: Which are the right descriptions of myopia treatment? |  |
| Eyeglasses can cure myopia. | 126 (29.8%) |
| Myopia can be cured by the laser surgery after adulthood. | 149 (35.2%) |
| *Myopia cannot be cured once it occurs. | 313 (74.0%) |
| *Pathological process of myopia (i.e. retina degeneration, elongated axial length) is reversible. | 51 (12.1%) |
| Myopia can be cured once visual acuity recovers to 1.0. | 77 (18.2%) |
| Q14: What is the correlation between classroom seating and myopia? |  |
| The seat in the first row is correlated with the development of myopia. | 71 (16.8%) |
| The seat in the last row is correlated with the development of myopia. | 41 (9.7%) |
| The seat on both sides is correlated with the development of myopia. | 44 (10.4%) |
| *The seat is not correlated with the development of myopia. | 309 (73.0%) |
| Q15: What kinds of behaviors do near work refer to? |  |
| *Reading | 365 (86.3%) |
| *Doing homework | 364 (86.1%) |
| *Using mobile phones | 406 (96.0%) |
| *Watching television | 266 (62.9%) |
| *Playing with building blocks | 107 (25.3%) |
| *Making homemade crafts | 143 (33.8%) |
| *Playing with piano | 164 (38.8%) |
| *Drawing | 224 (53.0%) |
| Q16: What kinds of activities do outdoor exercise include? |  |
| *Sports activities in the open air (i.e. running, playing football) | 407 (96.2%) |
| *Other activities in the open air (i.e. walking) | 376 (88.9%) |
| Sports activities in venues (i.e. playing table tennis, taekwondo, swimming) | 138 (32.6%) |
| *Field activities (i.e. mountaineering, climbing) | 389 (92.0%) |
| Unclear | 25 (0.6%) |

***:** the expected answers with right knowledge.

***Supplementary Table 4***. The correlation analysis of demographic information and parental knowledge score.

| **Variables** | **Simple linear regression** | | | **Multiple linear regression** | | |
| --- | --- | --- | --- | --- | --- | --- |
|  | **Weight coefficient** | **95% CI** | **P value** | **Weight coefficient** | **95% CI** | **P value** |
| Children’s age | -0.69 | -1.21, -0.17 | 0.009* | -0.60 | -1.12, -0.07 | 0.026* |
| Children’s gender (Male) | 0.88 | -2.25, 4.01 | 0.581 |  |  |  |
| Children’s number | 1.28 | -2.16, 4.73 | 0.465 |  |  |  |
| Place of residence (Urban) | 18.40 | 8.95, 27.86 | <0.001* | 9.52 | -0.36, 19.40 | 0.059 |
| Father’s age | -0.07 | -0.35, 0.22 | 0.653 |  |  |  |
| Mother’s age | -0.13 | -0.50, 0.24 | 0.497 |  |  |  |
| Father’s education | -2.90 | -4.47, -1.34 | <0.001* | 1.05 | -1.14, 3.24 | 0.347 |
| Mother’s education | -3.99 | -5.59, -2.39 | <0.001* | -2.17 | -4.42, 0.08 | 0.059 |
| Father’s occupation |  |  |  |  |  |  |
| Medicine and healthcare | 1.35 | -5.56, 8.25 | 0.702 |  |  |  |
| Physical labor | -16.36 | -25.85, -6.87 | 0.001* | -9.21 | -22.24, 3.83 | 0.166 |
| Mental labor | 6.69 | 2.53, 10.85 | 0.002* | 1.05 | -5.89, 7.99 | 0.767 |
| Free occupation | -7.29 | -12.89, -1.70 | 0.011* | -3.25 | -12.44, 5.93 | 0.486 |
| Mother’s occupation |  |  |  |  |  |  |
| Medicine and healthcare | 0.19 | -4.37, 4.76 | 0.934 |  |  |  |
| Physical labor | -12.39 | -23.57, -1.22 | 0.030* | 0.84 | -12.42, 14.10 | 0.901 |
| Mental labor | 4.03 | 0.58, 7.48 | 0.022* | 0.75 | -3.87, 5.36 | 0.750 |
| Free occupation | -5.43 | -10.12, -0.75 | 0.023* | 0.61 | -5.92, 7.14 | 0.854 |
| Myopic father | 5.87 | 2.55, 9.19 | 0.001* | 2.98 | -0.43, 6.38 | 0.086 |
| Myopic mother | 3.76 | 0.10, 7.42 | 0.044* | -0.06 | -3.79, 3.67 | 0.974 |
| Income | 3.62 | 2.24, 5.00 | <0.001* | 2.60 | 1.13, 4.07 | 0.001* |

*: significantly correlated with parental knowledge (p<0.05)

***Supplementary Table 5***. The information of children’s behaviors and its correlation with children’s onset of myopia.

| **Children’s behaviors** | **Total** | **Myopia group** | **Non-myopia group** | **P value**  **for univariate analysis** | **P value**  **for logistic analysis** | **OR** | **95%CI** |
| --- | --- | --- | --- | --- | --- | --- | --- |
| Daily time spent on |  |  |  |  |  |  |  |
| Sleeping |  |  |  | 0.002* | 0.945 | 1.02 | 0.58, 1.81 |
| <7h | 38 (9.0%) | 35(10.3%) | 2(4.8%) |  |  |  |  |
| 7-8h | 139 (32.9%) | 120(35.2%) | 6(14.3%) |  |  |  |  |
| 8-9h | 172 (40.7%) | 137(40.2%) | 24(57.1%) |  |  |  |  |
| 9-10h | 66 (15.6%) | 43(12.6%) | 10(23.8%) |  |  |  |  |
| >10h | 8 (1.9%) | 6(1.8%) | 0(0%) |  |  |  |  |
| Doing homework |  |  |  | <0.001* | 0.046* | 1.61 | 1.01, 2.58 |
| <1h | 23 (5.4%) | 8(2.3%) | 10(23.8%) |  |  |  |  |
| 1-2h | 58 (13.7%) | 39(11.4%) | 7(16.7%) |  |  |  |  |
| 2-4h | 132 (31.2%) | 110(32.3%) | 15(35.7%) |  |  |  |  |
| 4-8h | 141 (33.3%) | 124(36.4%) | 9(21.4%) |  |  |  |  |
| >8h | 65 (15.4%) | 60(17.6%) | 1(2.4%) |  |  |  |  |
| Outdoor activities |  |  |  | 0.173 |  |  |  |
| <0.5h | 111 (26.2%) | 85(24.9%) | 7(16.7%) |  |  |  |  |
| 0.5-1h | 157 (37.1%) | 132(38.7%) | 16(38.1%) |  |  |  |  |
| 1-1.5h | 103 (24.3%) | 85(24.9%) | 13(31.0%) |  |  |  |  |
| 1.5-2h | 37 (8.7%) | 28(8.2%) | 3(7.1%) |  |  |  |  |
| >2h | 15 (3.5%) | 11(3.2%) | 3(7.1%) |  |  |  |  |
| Electronic products |  |  |  | 0.131 |  |  |  |
| <0.5h | 77 (18.2%) | 60(17.6%) | 10(23.8%) |  |  |  |  |
| 0.5-1h | 126 (29.8%) | 104(30.5%) | 14(33.3%) |  |  |  |  |
| 1-1.5h | 71 (16.8%) | 56(16.4%) | 7(16.7%) |  |  |  |  |
| 1.5-2h | 48 (11.3%) | 42(12.3%) | 6(14.3%) |  |  |  |  |
| >2h | 99 (23.4%) | 79(23.2%) | 5(11.9%) |  |  |  |  |
| Daily frequency of |  |  |  |  |  |  |  |
| Taking a break after using eyes continuously for 40 minutes |  |  |  | <0.001* | 0.233 | 1.33 | 0.83, 2.14 |
| Always | 35 (8.3%) | 21(6.2%) | 9(21.4%) |  |  |  |  |
| Usually | 152 (35.9%) | 117(34.3%) | 20(47.6%) |  |  |  |  |
| Often | 56 (13.2%) | 49(14.4%) | 6(14.3%) |  |  |  |  |
| Seldom | 142 (33.6%) | 125(36.7%) | 7(16.7%) |  |  |  |  |
| Never | 31 (7.3%) | 29(8.5%) | 0(0%) |  |  |  |  |
| Lying down to do homework and read books |  |  |  | 0.090 |  |  |  |
| Always | 23 (5.4%) | 18(5.3%) | 3(7.1%) |  |  |  |  |
| Usually | 48 (11.3%) | 42(12.3%) | 1(2.4%) |  |  |  |  |
| Often | 52 (12.3%) | 44(12.9%) | 5(11.9%) |  |  |  |  |
| Seldom | 174 (41.1%) | 148(43.4%) | 17(40.5%) |  |  |  |  |
| Never | 113 (26.7%) | 89(26.1%) | 16(38.1%) |  |  |  |  |
| Keeping the distance between eyes and books over 30 centimeters when reading |  |  |  | <0.001* | 0.062 | 1.53 | 0.98, 2.39 |
| Always | 22 (5.2%) | 15(4.4%) | 5(11.9%) |  |  |  |  |
| Usually | 110 (26.0%) | 81(23.8%) | 18(42.9%) |  |  |  |  |
| Often | 104 (24.6%) | 84(24.6%) | 13(31.0%) |  |  |  |  |
| Seldom | 151 (35.7%) | 135(39.6%) | 3(7.1%) |  |  |  |  |
| Never | 29 (6.9%) | 26(7.6%) | 3(7.1%) |  |  |  |  |
| Keeping enough bright light when reading and writing |  |  |  | 0.396 |  |  |  |
| Always | 216 (51.1%) | 177(51.9%) | 19(45.2%) |  |  |  |  |
| Usually | 179 (42.3%) | 145(42.5%) | 20(47.6%) |  |  |  |  |
| Often | 18 (4.3%) | 14(4.1%) | 2(4.8%) |  |  |  |  |
| Seldom | 6 (1.4%) | 5(1.5%) | 0(0%) |  |  |  |  |
| Never | 1 (0.2%) | 0(0%) | 1(2.4%) |  |  |  |  |
| Doing Chinese eye exercises |  |  |  | 0.314 |  |  |  |
| Always | 45 (10.6%) | 36(10.6%) | 4(9.5%) |  |  |  |  |
| Usually | 115 (27.2%) | 97(28.4%) | 13(31.0%) |  |  |  |  |
| Often | 55 (13.0%) | 49(14.4%) | 4(9.5%) |  |  |  |  |
| Seldom | 127 (30.0%) | 111(32.6%) | 8(19.0%) |  |  |  |  |
| Never | 75 (17.7%) | 48(14.1%) | 13(31.0%) |  |  |  |  |
| Keeping a balanced nutrition diet |  |  |  | 0.646 |  |  |  |
| Always | 153 (36.2%) | 129(37.8%) | 11(26.2%) |  |  |  |  |
| Usually | 153 (36.2%) | 116(34.0%) | 22(52.4%) |  |  |  |  |
| Often | 74 (17.5%) | 61(17.9%) | 6(14.3%) |  |  |  |  |
| Seldom | 36 (8.5%) | 29(8.5%) | 2(4.8%) |  |  |  |  |
| Never | 7 (1.7%) | 6(1.8%) | 1(2.4%) |  |  |  |  |

*: significantly correlated with children’s myopia status (p<0.05)

***Supplementary Table 6***. Subgroups analysis of children’s behaviors before and after the outbreak of COVID-19 on the basis of parental knowledge.

|  | **Sleep** | **Homework** | **Outdoor activities** | **Electronic products** |
| --- | --- | --- | --- | --- |
| High knowledge | <0.001* | 0.034* | <0.001* | <0.001* |
| Poor knowledge | <0.001* | 0.657 | <0.001* | <0.001* |

*: significantly different between high and poor knowledge subgroups (p<0.05)
